# Hand hygiene compliance among healthcare workers before and after a CFIR-guided role-stratified intervention: a mixed-methods study in a tertiary hospital

**DOI:** 10.3389/fpubh.2026.1750206

**Published:** 2026-04-20

**Authors:** Na Li, Xin Guan, Rui Zhang, Juyuan Liu, Xiaolin Li

**Affiliations:** Department of Hospital Infection Control, Beijing Hospital, National Center for Gerontology, National Clinical Research Center for Gerontology, The Key Laboratory of Geriatrics of NHC, Institute of Geriatric Medicine, Chinese Academy of Medical Sciences, Beijing, China

**Keywords:** consolidated framework for implementation research (CFIR), hand hygiene, health personnel, healthcare-acquired INFECTIONs, infection control

## Abstract

**Introduction:**

This study aimed to evaluate hand hygiene compliance among healthcare workers of different roles, identify key barriers using the Consolidated Framework for Implementation Research (CFIR), and assess the effectiveness of a CFIR-guided stratified intervention in a tertiary hospital.

**Methods:**

An exploratory sequential mixed-methods study was conducted at Beijing Hospital, National Center of Gerontology, from December 2024 to September 2025. The observational phase included 9,767 non-announced direct observation hand hygiene observations and a survey of 275 staff (71 physicians, 173 nurses, 31 support staff), supplemented by 15 semi-structured interviews. A six-month CFIR-guided intervention was then implemented, incorporating role-specific training sessions, data-driven feedback, visual reminders, and position-based supervision tailored to physicians, nurses, and support staff. Outcomes included hand hygiene compliance and correctness, knowledge scores, and hospital-acquired infection (HAI) rates.

**Results:**

At baseline, compliance differed by role: nurses 81.6%, physicians 72.6%, and support staff 39.3% (*p* < 0.001). Knowledge scores were significantly lower among support staff (58.6 ± 9.4) than physicians and nurses (66.1 ± 6.9, *p* < 0.001). Qualitative interviews and CFIR-based surveys identified barriers including workflow constraints, insufficient supervision, inadequate training coverage, and knowledge gaps. Following the intervention, overall compliance increased from 74.9 to 85.4% (*p* < 0.001), with the largest improvement among support staff (39.3% vs. 56.0%, *p* = 0.002). Compliance improved across all “five moments,” particularly before patient contact (+18.5%, p < 0.001). HAI rates were lower from 3.05 per 1,000 patient-days to 1.66 per 1,000 patient-days (*p* = 0.002), with reductions observed in respiratory, urinary tract, bloodstream, and surgical site infections during the study period.

**Conclusion:**

Hand hygiene compliance varies substantially across healthcare worker roles, with support staff representing a critical gap. A CFIR-guided, stratified intervention was associated with increases in compliance and correctness, particularly at high-risk moments, and reduced HAI incidence, suggesting its potential value as a sustainable framework for hospital-wide infection prevention.

## Introduction

Hospital-acquired infections (HAIs) are among the most frequent and severe adverse events in healthcare settings. On average, approximately 1 in 10 hospitalized patients worldwide experiences an HAI, with even higher rates observed in intensive care units (ICUs) and in low- and middle-income countries (1). In the European Union and European Economic Area, over 3.5 million HAIs occur annually, leading to more than 90,000 deaths and imposing a burden exceeding that of influenza and tuberculosis (2). In long-term care facilities, a 12-month cumulative incidence of 124.2 HAIs per 100 residents (95% CI: 118.6–129.9) has been reported in a European multicenter cohort study (3). These infections not only increase morbidity and mortality, but also prolong hospital stays, elevate healthcare costs, and exacerbate the rise of antimicrobial resistance.

Hand hygiene is one of the most critical measures for preventing hospital-acquired infections, characterized by its simplicity, cost-effectiveness, and efficiency (4, 5). Contaminated hands are a leading pathway for nosocomial pathogen transmission, and proper handwashing and hand-rubbing can reduce catheter-related bloodstream infections. According to the *Notice on the Medical Quality Control Indicators for Hospital Infection Management (2024 Edition)* issued by the National Health Commission of China (6), for the first time, non-clinical support staff have been incorporated into the scope of hand hygiene monitoring. This marks a new stage in infection prevention and control, moving toward full staff participation. However, current studies have primarily focused on healthcare professionals, with a lack of systematic analyses grounded in implementation science. Despite its critical importance, disparities in hand hygiene compliance and knowledge are well-documented. Adherence rates vary widely—ranging from as low as 21% in some low-resource settings such as Ethiopia to highs of 84.3% in better-resourced countries like Australia (7). Self-reported compliance is often higher than what is observed, underscoring the risk of reporting bias (8). Numerous barriers to hand hygiene implementation have been identified: at the individual level, inadequate knowledge and negative attitudes toward hand hygiene are major hindrances (7); management-related factors include poor role modeling by supervisors, insufficient training, and weak planning (9); organizational and environmental constraints such as heavy workloads, poorly designed ward layouts, lack of equipment, and low-quality supplies further impede compliance (10).

Despite abundant evidence of these issues (11–13), previous studies often had limitations: many relied on self-reported data prone to bias, used cross-sectional designs without follow-up, or lacked theoretical grounding in implementation frameworks. Additionally, few studies have integrated both quantitative and qualitative methods to identify barriers while simultaneously testing structured interventions guided by implementation science. This study innovatively applies the Consolidated Framework for Implementation Research (CFIR) (14), combining quantitative observations with qualitative interviews to elucidate the mechanisms driving differences in hand hygiene behaviors across professional roles. In particular, we hypothesized that CFIR domains such as individual characteristics (knowledge and attitudes), inner setting (leadership engagement and feedback climate), intervention characteristics (perceived complexity), and implementation process (training coverage and supervision) would vary by professional role; qualitative interviews were used to identify role-specific barriers within these domains, and quantitative surveys were applied to assess their distribution and relative prominence across groups. In clinical practice—particularly in high-intensity departments such as surgery and intensive care—strict adherence to hand hygiene remains challenging due to time constraints, heavy workloads, and competing priorities (15–17). Some studies have discussed the possibility that incremental gains may plateau at higher levels of compliance (18–20). Support staff, who are in frequent contact with the ward environment, represent a critical link in preventing cross-contamination. Yet, related research remains relatively limited. This study integrates the World Health Organization (WHO) standard hand hygiene questionnaire and hospital infection surveillance data to evaluate differences in compliance across healthcare worker groups (21). It also aims to identify implementation barriers via CFIR-informed qualitative and quantitative assessments, and determine the effectiveness of a role-specific, CFIR-guided intervention in improving compliance and reducing hospital-acquired infection rates.

## Materials and methods

### Study design and subjects

This study adopted an exploratory sequential mixed-methods design, consisting of a cross-sectional observational phase and a quasi-experimental pre-post intervention phase without a parallel control group. The study was conducted at Beijing Hospital, National Center of Gerontology, between December 2024 and September 2025. The cross-sectional phase was carried out from December 2024 to March 2025, and the quasi-experimental intervention phase was implemented from April 2025 to September 2025. Participants were recruited from three surgical wards, three internal medicine wards, and intensive care units (ICU, RCU, NICU).

Hospital setting and hand hygiene infrastructure. The study wards comprised internal medicine wards (*n* = 6; respiratory medicine × 2, cardiology × 2, gastroenterology/endocrinology × 2), surgical wards (*n* = 6; general surgery × 2, orthopedics × 2, vascular surgery × 2), and critical care units (*n* = 3; ICU × 1, RCU × 1, NICU × 1). In the internal medicine and surgical wards, rooms were multi-patient rooms (2–4 beds per room), with approximately 40–43 beds per internal medicine ward and 40–48 beds per surgical ward. In the critical care units, rooms were single- or double-bed rooms, with approximately 10–17 beds per unit. The participating wards remained structurally unchanged during the study period, and patient-days were derived from the hospital's electronic inpatient management system for the corresponding pre- and post-intervention periods. Hand hygiene facilities were configured under a unified policy across wards: all physicians, nurses, and support staff were provided with portable alcohol-based hand rub; fixed alcohol-based hand rub dispensers were installed at each room entrance and inside each patient room; alcohol-based hand rub was available on each treatment cart and near major monitoring equipment; and sinks with running water were available in patient rooms and at nursing and physician stations. Key differences by ward type were as follows: in general wards (internal medicine and surgery), one alcohol-based hand rub point was placed in each patient room and an additional dispenser was placed in the corridor area between adjacent rooms; in critical care units (ICU/RCU/NICU), alcohol-based hand rub was placed at the foot of each bed to ensure point-of-care availability.

Eligible participants included physicians, nurses, and support staff (i.e., cleaners and caregivers) employed for ≥3 months. In this study, support staff referred specifically to cleaners and caregivers whose routine duties required entering patient rooms and having contact with patients or the patient environment. Distribution and logistics personnel were not included, as they generally did not enter clinical care areas and did not routinely encounter the full set of hand hygiene opportunities defined by the World Health Organization (WHO) “Five Moments for Hand Hygiene,” which include (1) before touching a patient, (2) before clean/aseptic procedures, (3) after body fluid exposure risk, (4) after touching a patient, and (5) after touching patient surroundings (22). Exclusion criteria were interns, visiting trainees, and staff unwilling to participate.

The study protocol was approved by the hospital's ethics committee (Approval No. 2024BJYYEC-KY332-02).

### Data collection

#### Questionnaire survey

The WHO Hand Hygiene Knowledge and Perception Questionnaire, an internationally recognized tool, is designed to assess healthcare workers' professional knowledge and perceptions of hand hygiene (23). The Chinese version of the WHO Hand Hygiene Knowledge and Perception Questionnaire demonstrated a scale-level content validity index (S-CVI/Ave) of 0.90, with an overall Cronbach's α of 0.85 and dimension-specific α coefficients ranging from 0.76 to 0.89. Prior to distribution, items were reviewed for linguistic clarity, and trained investigators provided on-site clarification when necessary, particularly for support staff with lower educational backgrounds.

The WHO Hand Hygiene Observation Form, a WHO-recommended standard observation form, was used to record department information, professional category, compliance with the Five *Moments for Hand Hygiene* (compliance rate = number of hand hygiene actions / number of hand hygiene opportunities × 100), and correctness (24). Hand hygiene practices were assessed through non-announced direct observation conducted by trained observers during routine clinical activities. Observations were based on the World Health Organization (WHO) hand hygiene technique, with emphasis on adequate coverage of all hand surfaces. For alcohol-based hand rub, hand rubbing was considered appropriate when performed until hands were dry, typically corresponding to approximately 20–30 s according to WHO guidance. Assessment focused on completeness of surface coverage rather than strict adherence to a predefined step sequence or fixed time threshold. The same observation criteria were applied consistently during both the pre-intervention and post-intervention periods. Correctness rate was defined as the proportion of hand hygiene actions performed with adequate technique and complete surface coverage.

Questionnaires were distributed both electronically and on paper. Paper questionnaires were collected on-site by trained investigators, who also checked for completeness. Data were double-entered, with < 1% missing values imputed by mean substitution for descriptive analyses; given the very low proportion of missing data, no sensitivity analysis was performed.

#### On-site non-announced direct observation

Six full-time infection control professionals were trained and certified as observers to standardize observation procedures. Training covered: (1) Recognition of the five moments for hand hygiene (WHO 2009 Guidelines: before patient contact, before aseptic procedures, after body fluid exposure, after patient contact, after contact with the patient environment) (21). (2) Criteria for correct hand hygiene performance, including full coverage of key steps (palms, backs of hands, interdigital spaces, backs of fingers, thumbs, fingertips, and wrists). (3) Standardized completion of observation forms, reinforced through scenario simulations. Following training, observers conducted a pilot observation of 20 healthcare workers in a non-study ward. Inter-observer reliability testing yielded a Kappa value of 0.82, indicating good agreement. To minimize observer drift over the 10-month study period, several ongoing quality control measures were implemented. All observers were full-time infection control professionals with more than 5 years of experience and were trained strictly according to the WHO “My 5 Moments for Hand Hygiene” framework. Consensus meetings were held to discuss ambiguous cases and reinforce standardized judgment criteria. In addition, periodic paired spot-check observations were conducted by a senior infection control supervisor to verify recording Correctness rate, and discrepancies were resolved immediately through discussion. Weekly data logic checks were also performed to identify and correct implausible entries. The observer team remained stable throughout the study period.

During formal observation, a non-announced direct observation approach was used. Healthcare workers were aware that hand hygiene practices might be monitored as part of routine infection prevention activities; however, they were not informed of the specific timing, location, observers, or detailed study hypotheses. Nine wards were sampled across different shifts, focusing on high-frequency clinical periods. Each session targeted one staff member for ≤ 20 min, recording all hand hygiene actions during medical, nursing, or cleaning procedures.

#### Observation workload and scheduling

Hand hygiene observations were conducted between December 2024 and September 2025, including a 4-month pre-intervention phase and a 6-month intervention phase. A total of 9,767 hand hygiene opportunities were recorded during this 10-month period, corresponding to an average of approximately 33 observations per day across all observers, or 7-8 observations per observer per day (assuming 22 working days per month). Observers were assigned using a stratified rotation strategy: the 15 wards were grouped into internal medicine (*n* = 6), surgery (*n* = 6), and critical care units (*n* = 3), with observers working in pairs and rotating across groups every 2 months to reduce observer bias. Observation time points were selected using a random scheduling approach and covered high-activity clinical periods, including morning care, physician rounds, afternoon procedures, and selected weekend shifts. Observations were conducted discreetly without interfering with routine clinical workflow.

#### Hospital infection surveillance

Hospital-acquired infection (HAI) data were extracted from the hospital infection surveillance system. All HAIs included in this study were confirmed cases meeting standardized diagnostic criteria for hospital-acquired infection (onset ≥48 h after admission and fulfilling clinical and/or microbiological criteria). Overall HAI incidence included all validated hospital-acquired infections, not limited to device-associated infections or surgical site infections. Community-acquired infections and non-validated events were excluded. Infection incidence was expressed as the number of cases per 1,000 patient-days. For each observation period, the total number of patient-days and infection cases were extracted to calculate incidence rates. Data were extracted for two observation periods: the pre-intervention baseline and the post-intervention phase. The surveillance data were used to analyze changes in HAI incidence and infection type distribution before and after implementation of the CFIR-guided hand hygiene improvement program.

#### Semi-structured interviews

A CFIR-based semi-structured interview guide was developed based on prior observations and a review of relevant literature. The guide encompassed the five CFIR domains—intervention characteristics, outer setting, inner setting, individual characteristics, and implementation process—to systematically identify barriers to hand hygiene practices. The draft guide was reviewed and refined by three experts in infection control, nursing management, and public health to ensure scientific rigor and practical relevance.

The study began with qualitative interviews to identify major barrier themes, followed by quantitative surveys to assess their prevalence across different staff roles. In the qualitative phase, 15 representative staff members (five physicians, five nurses, and five support staff) were purposively selected to ensure diversity in roles, responsibilities, and work contexts. All interviews were audio-recorded with participants' permission and transcribed verbatim. A coding framework was developed based on the Consolidated Framework for Implementation Research (CFIR), using a deductive approach to structure initial codes while allowing inductive identification of emergent themes. Two researchers independently coded the transcripts, and discrepancies were resolved through discussion until consensus was reached. Monthly analytic meetings were conducted to refine the codebook and ensure consistent interpretation across cases. Thematic analysis was then applied to synthesize key barrier themes from the coded data. Triangulation was performed by comparing qualitative themes with quantitative findings. For example, support staff's reported dissatisfaction with training was consistent with the higher proportion identifying “insufficient training coverage” as a barrier (70.5%) and with significantly lower knowledge scores compared with physicians and nurses (*p* < 0.001). Similarly, physicians' reports of workflow-related time pressure aligned with the lowest compliance observed before patient contact. Sample size was determined according to the principle of thematic saturation; thematic stability was observed after approximately 13 interviews, with no substantially new barrier categories emerging in the final interviews. However, given the heterogeneity of professional roles and departments, the findings should be interpreted as capturing the principal barrier patterns within this setting rather than exhaustive coverage of all possible perspectives. Findings from the qualitative phase informed the development of questionnaire items for the subsequent quantitative survey within the CFIR framework.

The interview guide covered five CFIR domains: intervention characteristics, inner setting, outer setting, individual characteristics, and implementation process. During analysis, key constructs within each domain were identified and coded. For example, within the “individual characteristics” domain, knowledge, and beliefs about hand hygiene were examined; within the “inner setting” domain, feedback culture and workflow compatibility were assessed; and within the “implementation process” domain, training adequacy and supervision mechanisms were analyzed. Themes were mapped to these constructs to ensure conceptual alignment with the CFIR framework.

## Intervention

The intervention aimed to improve both compliance and correctness of hand hygiene across all hospital staff categories, with particular emphasis on high-risk moments, including before patient contact, and before aseptic procedures, ultimately aiming to reduce hospital-acquired infection (HAI) rates. The program was implemented over 6 months (April–September 2025), incorporating dynamic feedback to facilitate continuous improvement. Interventions were structured according to the CFIR framework and included both role-specific and system-level components. The core elements are summarized below, with additional implementation details provided in Supplementary Table 1. Key elements included: (1) Position-based stratified management: tailored strategies for physicians, nurses, and support staff, with joint supervision by medical staff in departments heavily reliant on support staff. (2) Data-driven feedback: monthly audit-and-feedback reports generated from observational data, summarizing compliance and correctness rates by ward and professional group. Predefined performance thresholds (≥80% acceptable; 60%−79% improvement needed; < 60% targeted corrective action) guided subsequent improvement measures. (3) CFIR-structured design: interventions grounded in organizational and behavioral science evidence, with barriers identified through qualitative interviews and questionnaire analysis. (4) Visual aids and training: workflow charts, reminder cards, and role-specific training sessions delivered monthly by infection control professionals (30–45 min per session), with content adapted to staff education levels and focused on technique demonstration and scenario-based discussion. (5) Cross-departmental supervision: joint oversight by medical, nursing, and logistics teams to ensure coordinated implementation across wards. (6) PDCA cycle and continuous quality improvement: iterative evaluation and adjustment conducted monthly based on performance data and supervisory review. Intervention fidelity was monitored through attendance records, documentation of feedback dissemination, and regular implementation meetings. An example of the structured monthly audit-and-feedback report template is provided in Supplementary Table 2.

### Sample size calculation

The sample size calculation applied only to the questionnaire survey assessing hand hygiene knowledge and attitudes. Based on a pilot survey, the expected proportion of the primary variable was conservatively set at 50% (*P* = 0.5). With a confidence level of 95% (*Z* = 1.96) and a margin of error of 0.06 (*d* = 0.06), the required sample size was calculated using the cross-sectional formula: *n* = *Z*^2^ × P × (1 – P) / d^2^ This yielded a minimum required sample size of 267 participants. Considering potential non-response or invalid questionnaires, a total of 275 healthcare workers were ultimately included, meeting the analytic requirement. Hand hygiene observation data and hospital-acquired infection surveillance data were derived from routine quality monitoring and infection surveillance systems. No *a priori* power calculation was performed for pre–post comparisons of compliance or infection outcomes, as these analyses were designed to describe changes within a real-world quality improvement framework rather than to test a predefined effect size.

### Statistical analysis

Data were entered using Excel and analyzed with SPSS version 25.0 (IBM, New York, USA). Categorical variables were expressed as frequencies and percentages, and continuous variables as mean ± standard deviation. Group comparisons were performed using ANOVA with Bonferroni correction. Compliance and correctness rates were compared using chi-square tests. A two-sided *P* < 0.05 was considered statistically significant. Because individual identifiers were not recorded for ethical reasons, clustering of hand hygiene opportunities within individuals or wards could not be explicitly modeled. Therefore, the assumption of independence between observations may not be fully satisfied, and statistical comparisons were interpreted cautiously, primarily to describe trends rather than to establish definitive inferential conclusions. Monthly HAI rates were plotted to visually illustrate temporal trends; however, formal interrupted time series or time-series regression modeling was not performed. Comparisons were primarily conducted between aggregated pre- and post-intervention periods.

## Results

### Hand hygiene knowledge and perception

Of the 300 distributed questionnaires, 275 valid responses were collected (response rate: 91.7%). Participants demonstrated a generally positive perception of hand hygiene, with a mean attitude score of 89.4 ± 5.2. However, knowledge levels were moderate, with a mean score of 64.2 ± 8.7. One-way ANOVA indicated significant differences in knowledge across job categories [F(2,272) = 10.37, *p* < 0.001]. Bonferroni tests showed no significant difference between physicians (67.5 ± 7.1) and nurses (65.3 ± 6.8) (*p* = 0.072), but the combined physician-nurse group (mean 66.1 ± 6.9) scored significantly higher than support staff (58.6 ± 9.4, *p* < 0.001) (Table 1).

**Table 1:** Knowledge and perception scores of hand hygiene among healthcare workers by professional category (*n* = 275).

Position	Sample size (*n*)	Attitude score (mean ±SD)	Knowledge score (mean ±SD)
Physicians	71	90.1 ± 4.8	67.5 ± 7.1
Nurses	173	89.2 ± 5.4	65.3 ± 6.8
Support staff	31	88.3 ± 5.6	58.6 ± 9.4

Differences in knowledge scores among positions were statistically significant [F(2,272) = 10.37, *p* < 0.001]. Physicians and nurses scored significantly higher than support staff (Bonferroni correction, *p* < 0.001). Attitude scores were generally high, with no significant differences among positions (*p* > 0.05).

### Compliance and correctness by job category

Among 9,767 observed hand hygiene opportunities, the overall compliance rate was 74.9%, and the correctness rate was 81.4%. Compliance was highest among nurses (81.6%), followed by physicians (72.6%), while support staff achieved only 39.3%. Correctness rates followed a similar pattern: nurses 89.0%, physicians 83.5%, and support staff 45.2%. These differences were statistically significant (χ^2^ = 912.73, *p* < 0.001) (Table 2).

**Table 2:** Hand hygiene compliance and correctness among physicians, nurses, and support staff (*N* = 9,767 observations).

Position	Observations (*n*)	Compliance rate % (95% CI)	Correctness rate % (95% CI)
Physicians	2,443	72.6 (71.0–74.1)	83.5 (82.2–84.8)
Nurses	5,978	81.6 (80.6–82.6)	89.0 (88.2–89.8)
Support staff	946	39.3 (36.2–42.4)	45.2 (42.0–48.4)

Compliance rate = number of compliant hand hygiene actions / number of observed opportunities. Correctness rate = number of correctly performed actions / number of compliant actions. Differences among positions were statistically significant (χ^2^ = 912.73, df = 2, *p* < 0.001). CI, confidence interval.

### Compliance across the five WHO “five moments”

Compliance varied significantly across the WHO-defined “five moments” (χ^2^ = 95.071, df = 4, *p* < 0.001). The highest compliance was observed after exposure to blood or body fluids (84.97%), followed by before aseptic procedures (79.98%), after patient contact (75.04%), and after contact with the patient's surroundings (65.02%). The lowest compliance was recorded before patient contact (50.03%), which was significantly lower than all other moments (*p* < 0.01) (Table 3).

**Table 3:** Compliance with hand hygiene across the five WHO-defined moments (%).

Indication	Observations	Compliance rate (%)
After exposure to blood or body fluids	946	84.97
Before aseptic procedures	1,150	79.98
After patient contact	2,540	75.04
After contact with patient surroundings	2,688	65.02
Before patient contact	2,443	50.03

Chi-square test showed significant differences in compliance rates across indications (χ^2^ = 95.071, df = 4, *p* < 0.001). Pairwise comparisons indicated that compliance before patient contact was significantly lower than at other indications (*p* < 0.01).

### Barrier analysis based on CFIR

Semi-structured interviews yielded 15 valid transcripts covering physicians, nurses, support staff, and cleaners. Line-by-line coding and thematic synthesis identified three representative themes reflecting frontline challenges in practice: (1) “Too busy to manage” (Physician, ID03): during high-intensity or emergency situations, staff sometimes touched the next patient immediately after glove removal, indicating a mismatch between workflow and clinical rhythm. (2) “No one notices if I wash” (Caregiver, ID12): in the absence of supervision and feedback, motivation declined, leading to skipped steps. (3) “Training feels perfunctory” (Cleaner, ID15): video-based training was insufficient for staff with lower educational backgrounds, highlighting the need for more practical, hands-on approaches (e.g., live demonstrations).

The structured questionnaire consisted of WHO-based knowledge and attitude items that were translated and culturally adapted into Chinese. In addition, two open-ended questions were included to explore perceived barriers to hand hygiene implementation. Thematic analysis of open-ended questionnaire responses and interview transcripts identified the following barriers, which were subsequently mapped to CFIR domains: (1) Intervention Characteristics (Process Complexity): 42.3% of participants perceived hand hygiene steps as overly complicated, especially during peak clinical workloads. (2) Inner Setting (Lack of Supervision and Feedback): 57.1% reported that hospitals lacked effective feedback mechanisms, hindering continuous compliance. (3) Implementation Process (Training Coverage): only 48.9% had received systematic hand hygiene training; coverage was markedly lower among support staff than physicians and nurses, suggesting uneven distribution of training resources. (4) Individual Characteristics (Perceived Knowledge Gaps): knowledge-related barriers were frequently mentioned in qualitative data. Quantitative knowledge scores, presented in Table 1, further demonstrated significantly lower knowledge among support staff (*p* < 0.001), reflecting weaker cognitive foundations for execution. (5) Outer Setting (Patient and Caregiver Influence): 29.8% reported interference from caregivers (e.g., interrupting procedures, touching medical surfaces), likely related to their limited awareness of hand hygiene and infection risks (Table 4). Identified themes were systematically mapped to corresponding CFIR constructs to ensure conceptual alignment. The CFIR framework was used as an analytic classification framework rather than as a quantitative measurement scale. For example, insufficient training coverage was categorized under the “implementation process” domain, while knowledge gaps were mapped to “individual characteristics.” Workflow-related time pressure was classified within the “inner setting” domain, reflecting structural and organizational constraints influencing hand hygiene behavior.

**Table 4:** Barriers to hand hygiene compliance based on the CFIR framework (*N* = 275).

CFIR domain	Overall barrier proportion (%)	Physicians & nurses (%)	Support staff (%)
Intervention characteristics	42.3	38.1	52.6
Inner setting	57.1	60.2	48.3
Implementation process	51.1	27.6	70.5
Individual characteristics (Perceived knowledge gaps)	46.9	39.8	68.4
Outer setting	29.8	31.5	25.7

^*^Indicates a large and practically meaningful difference between groups. ^**^Indicated Knowledge scores compared using t-test, with statistically significant differences (*p* < 0.001). CFIR, Consolidated Framework for Implementation Research.^***^The proportion reported under “Individual Characteristics” reflects the frequency of knowledge-related barriers identified through qualitative data. Quantitative knowledge scores are presented separately in Table 1.

Representative quotations further illustrate these barriers. A support staff member (ID15) stated: “The training sessions feel perfunctory, and the videos are difficult to understand for people with lower educational backgrounds.” A physician (ID03) described workflow pressure: “During busy rounds, after removing gloves, I sometimes move directly to the next patient without performing hand hygiene because everything happens so quickly.”

### Hand hygiene compliance and device-associated infection rates

In internal medicine, hand hygiene compliance was 68.9%, with an infection rate of 1.81 per 1,000 patient-days. In surgical wards, compliance was 70.7% and the infection rate was 0.63 per 1,000 patient-days. In intensive care units, compliance was 71.1%, but the infection rate was highest at 2.35 per 1,000 patient-days (Table 5).

**Table 5:** Hand hygiene compliance and device-associated infection rates.

Department	Compliance rate (%)	Device-associated infection rate (cases/1,000 patient-days)
Internal medicine	68.93	1.81
Surgery	70.65	0.63
Intensive care unit (ICU)	71.05	2.35

Data source—Hospital Infection Surveillance System.

### Changes in hospital-acquired infection rates and types before and after the intervention

**Figure 1:**
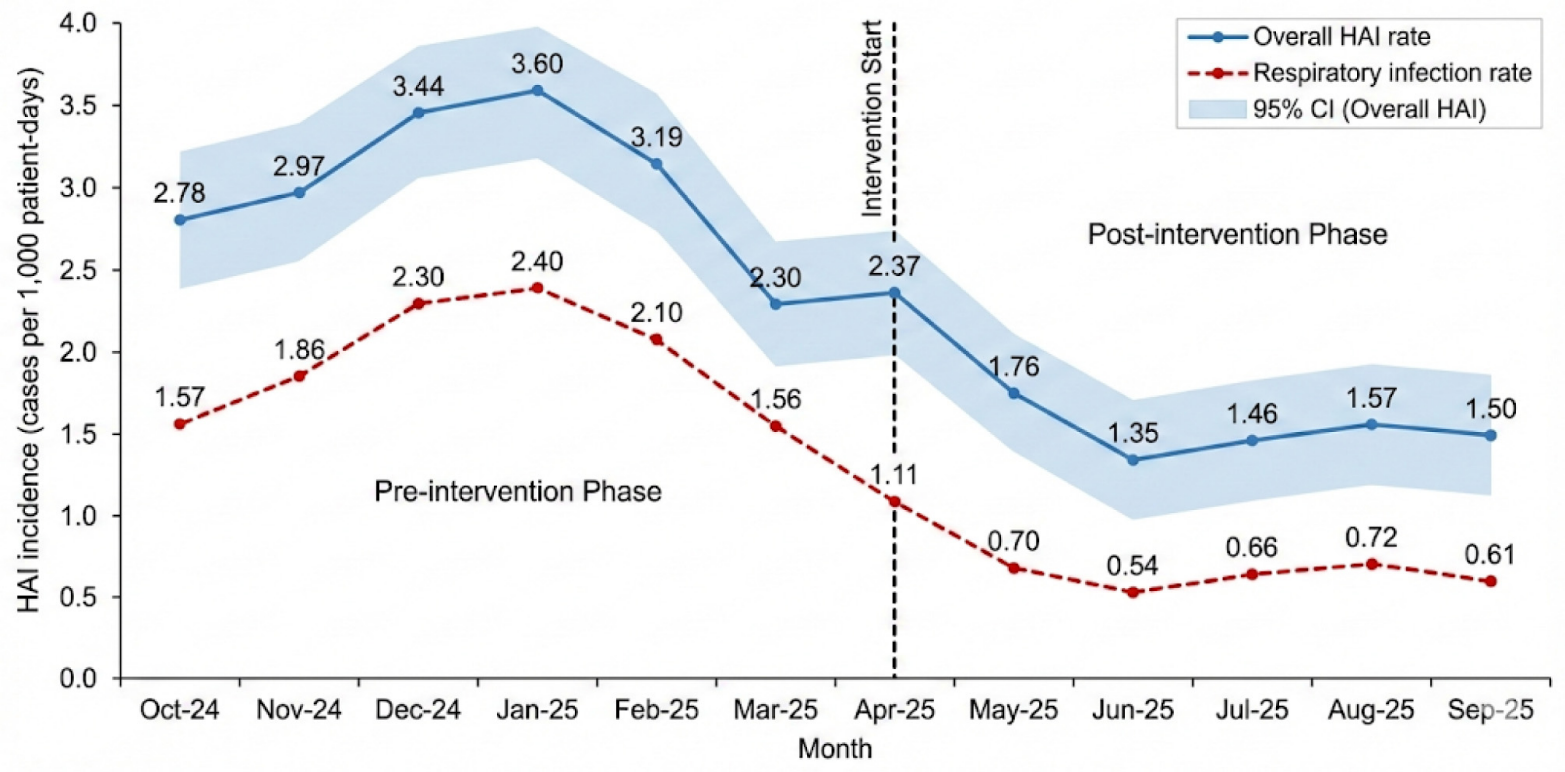
Monthly hospital-acquired infection (HAI) incidence before and after the CFIR-guided intervention. Infection rates are expressed as cases per 1,000 patient-days and include overall HAI rate, respiratory infections, urinary tract infections, bloodstream infections, and surgical site infections (SSI). The vertical dashed line indicates the initiation of the intervention (April 2025). Data are displayed as monthly rates to illustrate temporal patterns. This figure provides a descriptive visualization of infection trends and does not represent a formal interrupted time series analysis.

Following the 6-month CFIR-guided intervention, the overall HAI rate decreased from 3.05 per 1,000 patient-days to 1.66 per 1,000 patient-days (*p* < 0.001), corresponding to an absolute reduction of 1.39 cases per 1,000 patient-days and a relative reduction of approximately 45.6% (Table 6; Figure 1). The overall HAI category represents all confirmed hospital-acquired infections recorded in the surveillance system during each period. The corresponding numbers of infection cases and patient-days are presented in Table 6 to clarify the derivation of incidence rates.

**Table 6:** Changes in hospital-acquired infection (HAI) incidence per 1,000 patient-days before and after intervention.

Infection category	Pre-intervention cases (*n*)	Pre-intervention patient-days	Incidence per 1,000 patient-days (95% CI)	Post-intervention cases (*n*)	Post-intervention patient-days	Incidence per 1,000 patient-days (95% CI)	*P*-value
Respiratory infection	142	72,057	1.97 (1.64–2.29)	63	87,536	0.72 (0.54–0.90)	< 0.001
Urinary tract infection	12	72,057	0.17 (0.07–0.26)	13	87,536	0.15 (0.07–0.23)	0.742
Bloodstream infection	38	72,057	0.53 (0.36–0.70)	36	87,536	0.41 (0.27–0.55)	0.284
Surgical site infection (SSI)	28	72,057	0.39 (0.24–0.53)	31	87,536	0.35 (0.23–0.48)	0.698
Overall HAI	220	72,057	3.05 (2.64–3.45)	145	87,536	1.66 (1.39–1.93)	< 0.001

Infection rates were calculated as “number of infections per month / inpatient days × 1,000.” Chi-square test or *t*-test was used for analysis.

Data source: Hospital Infection Surveillance System, with definitions standardized according to the National Nosocomial Infection Management Guidelines.

Among infection subtypes, respiratory infections showed the largest reduction, decreasing from 1.97 to 0.72 per 1,000 patient-days (*p* < 0.001), representing an absolute reduction of 1.25 per 1,000 patient-days and a relative reduction of approximately 63.5%. In contrast, changes in urinary tract infections (0.17 per 1,000 patient-days vs. 0.15 per 1,000 patient-days; absolute reduction 0.02; relative reduction 11.8%; *p* = 0.742), bloodstream infections (0.53 per 1,000 patient-days vs. 0.41 per 1,000 patient-days; absolute reduction 0.12; relative reduction 22.6%; *p* = 0.284), and surgical site infections (0.39 per 1,000 patient-days vs. 0.35 per 1,000 patient-days; absolute reduction 0.04; relative reduction 10.3%; *p* = 0.698) were not statistically significant (Table 6; Figure 1). After Bonferroni correction for multiple comparisons across infection subtypes, the reduction in respiratory infections remained statistically significant, whereas changes in other infection categories did not meet the adjusted significance threshold (Table 6).

### Compliance and correctness by job category before and after intervention

**Figure 2:**
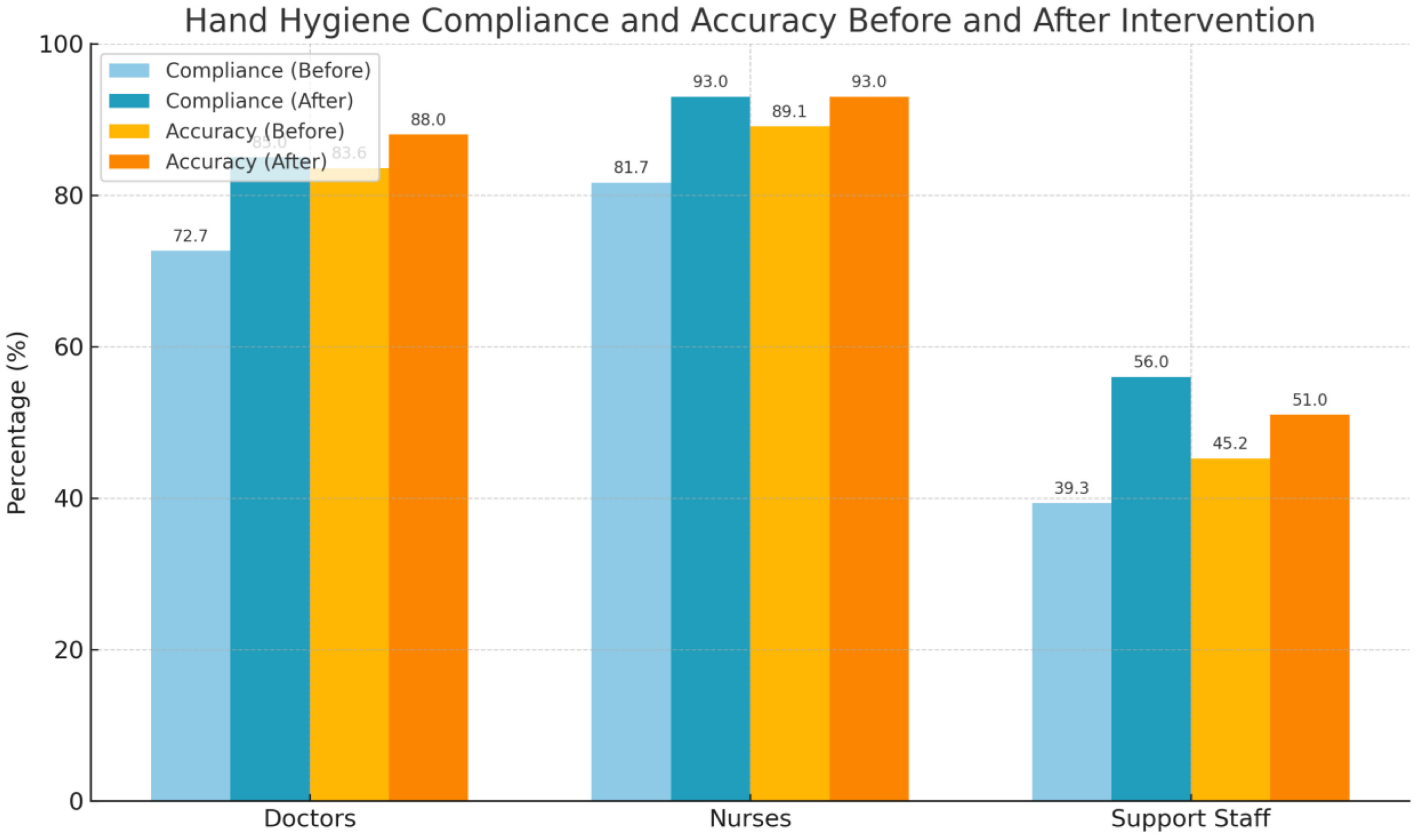
Compliance and correctness rates before and after intervention by job category. Data are expressed as percentages. χ^2^ test was used for comparisons.

Post-intervention, compliance and correctness improved significantly across all occupational groups. Physicians' compliance increased from 72.7 to 85.0% (*p* = 0.001), and correctness from 83.6 to 88.0% (*p* = 0.045). Nurses' compliance improved from 81.7 to 93.0% (*p* = 0.001), and correctness from 89.1 to 93.0% (*p* = 0.030). Support staff demonstrated the most substantial relative improvement in compliance (39.3 vs. 56.0%, *p* = 0.002), though the increase in correctness (45.2% vs. 51.0%) was not statistically significant (*p* = 0.060). Overall, compliance rose from 74.9 to 85.4% (*p* < 0.001), and correctness from 83.2 to 88.3% (*p* = 0.012) (Table 7; Figure 2). The distribution of observed hand hygiene opportunities by professional role and ward category did not differ significantly between the pre- and post-intervention periods (Supplementary Table 3), indicating structural comparability of sampling across phases. In terms of frequency, the average number of hand hygiene actions per person per day increased significantly: physicians from 6.3 ± 1.2 to 9.1 ± 1.4, nurses from 10.7 ± 1.5 to 13.4 ± 1.6, and support staff from 3.1 ± 1.0 to 6.2 ± 1.3 (all *p* < 0.001) (Table 8).

**Table 7:** Compliance and correctness of hand hygiene before and after intervention by job category.

Position	Indicator	Pre-*intervention* (%)	Post-intervention (%)	*p*-value
Physicians	Compliance	72.67	85.00	0.001
	Correctness rate	83.58	88.00	0.045
Nurses	Compliance	81.68	93.00	0.001
	Correctness rate	89.09	93.00	0.030
Support staff	Compliance	39.34	56.00	0.002
	Correctness rate	45.24	51.00	0.060
**Total**	Compliance	74.90	85.40	< 0.001
	Correctness rate	83.16	88.34	0.012

Compliance rate = number of compliant hand hygiene actions / number of observations. Correctness rate = number of correctly performed actions / number of compliant actions. *p*-values calculated using chi-square test.

**Table 8:** Daily hand hygiene frequency before and after intervention among different professional groups.

Position	Pre-intervention Mean (times/person/day)	Post-intervention Mean (times/person/day)	*p-*value
Physicians	6.3 ± 1.2	9.1 ± 1.4	< 0.001
Nurses	10.7 ± 1.5	13.4 ± 1.6	< 0.001
Support staff	3.1 ± 1.0	6.2 ± 1.3	< 0.001

Method: Hand hygiene execution frequency was collected through continuous non-announced direct observation observation. Observation targets were on-duty healthcare workers, and the data were converted into “times per person per day” based on actual working hours. Calculation formula: total number of observed hand hygiene actions ÷ number of observed person-days.

### Compliance across the five key moments post-intervention

Compliance improved across all five key moments after the intervention (Supplementary Figure S1). The greatest gain was observed before patient contact (50.1% vs. 68.6%, +18.5%, *p* < 0.001). Improvements were also evident after contact with the patient's surroundings (64.9% vs. 78.2%, +13.3%), after patient contact (75.1% vs. 86.9%, +11.8%), before aseptic procedures (79.9% vs. 90.1%, +10.2%), and after exposure to body fluids (85.0% vs. 92.6%, +7.6%) (Table 9).

**Table 9:** Compliance with hand hygiene at the five WHO-defined moments before and after intervention.

Moment category	Pre-intervention observations	Pre compliance (%)	Post-intervention observations	Post compliance (%)	Absolute improvement (%)
Before patient contact	2,443	50.10	3,600	68.60	+18.50
Before aseptic procedure	1,150	79.90	1,350	90.10	+10.20
After body fluid exposure	946	85.00	1,050	92.60	+7.60
After patient contact	2,540	75.10	3,100	86.90	+11.80
After contact with patient surroundings	2,288	64.90	2,114	78.20	+13.30

Improvements in compliance at all five moments were statistically significant (χ^2^ test, *p* < 0.001).

### Multivariable analysis of HAI incidence

To assess whether the observed changes in HAI rates were independent of variations in patient risk structure, a multivariable Poisson regression model was constructed. The model adjusted for monthly case mix index (CMI), surgical volume, device utilization rates (urinary catheter, central venous catheter, ventilator), and the proportion of ICU patient-days, with total patient-days included as an offset variable. After adjustment, the post-intervention period remained significantly associated with lower overall HAI incidence (adjusted IRR 0.62, 95% CI 0.50–0.77, *p* < 0.001) (Supplementary Table 4).

## Discussion

This CFIR-guided mixed-methods study revealed several key findings. Compliance and correctness differed significantly by professional role, with nurses performing best, physicians intermediate, and support staff lowest. Compliance was particularly poor before patient contact, one of the most critical infection-prevention moments. Barriers included insufficient training coverage, inadequate feedback, workflow constraints, and limited awareness among non-professional staff. Importantly, a 6-month stratified intervention was associated with improvements in compliance and correctness across all groups and at all five WHO-defined moments, with the greatest relative improvement among support staff and before patient contact. These gains were temporally associated with a reduction in overall HAI incidence. After adjustment for multiple comparisons, only respiratory infections remained statistically significant among infection subtypes, whereas reductions in other categories did not meet the corrected significance threshold. Collectively, these findings suggest that components targeting opportunity recognition, workflow integration, and performance feedback were more plausibly associated with improvements in compliance, whereas technique correctness—particularly among support staff—may require more intensive or sustained training efforts.

A key observation was that the baseline compliance rate of 74.9% is consistent with international data. Mouajou et al. (18), in a systematic review, reported a pooled compliance average of approximately 70%, with nurses consistently outperforming physicians, which mirrors our findings. Zou et al. (25) similarly documented a compliance rate of 71.4% among ICU staff in China, again noting superior performance by nurses. The persistence of such patterns across diverse contexts suggests that occupational role remains a stable determinant of adherence, shaped by differences in education, professional culture, and accountability.

Another important finding of this study was the particularly low compliance before patient contact (50.1%), which corroborates reports that healthcare workers often prioritize self-protection over patient safety. Issa et al. (19) and Wang et al. (20) both demonstrated markedly higher adherence after exposure to body fluids compared with pre-contact opportunities. This behavioral asymmetry underscores a systemic vulnerability, as inadequate hand hygiene before patient contact directly undermines infection prevention. Notably, the present intervention was designed to address this weakness, and the 18.5% increase in compliance reflects a meaningful improvement in this setting.

In addition, the barrier analysis deepens current understanding of role-specific challenges. Consistent with Yang et al. (27), insufficient feedback and limited training were identified as major barriers. The present study, however, further demonstrated that these barriers were disproportionately concentrated among support staff, with 70.5% reporting inadequate training compared with 27.6% of physicians and nurses. However, because formal measurement invariance testing of the knowledge and perception questionnaire across professional groups was not performed, between-group comparisons—particularly those involving support staff—should be interpreted with caution. Most previous studies have focused on professional staff, overlooking non-professional personnel despite their frequent contact with patient environments (25–27, 29). These findings are in line with Li et al. (28), who showed that targeted interventions among cleaning staff was associated with improvements in compliance significantly. Collectively, the evidence reinforces the importance of incorporating support staff into infection control programs, a principle reflected in recent Chinese national standards (6) mandating their inclusion in hand hygiene monitoring. Notably, although compliance among support staff improved significantly after the intervention, correctness did not reach statistical significance, indicating that improvements in opportunity recognition may have preceded improvements in technical execution.

Furthermore, the reduction in HAI incidence following the intervention is consistent with earlier studies while providing greater specificity. Han et al. (17) reported a reduction in the incidence of healthcare-associated infections from 1.10% to 0.91% after a 4-year multimodal hand hygiene intervention, suggesting a potential association between improved hand hygiene compliance and infection control outcomes, and Demirel et al. (30) demonstrated sustained declines after implementing PDCA cycle-driven improvements. The current study was consistent with prior observations and documented decreases across specific infection types: bloodstream infections declined from 0.53 to 0.41 per 1,000 patient-days, respiratory infections from 1.97 to 0.72 per 1,000 patient-days, urinary tract infections from 0.17 to 0.15 per 1,000 patient-days, and surgical site infections from 0.39 to 0.35 per 1,000 patient-days. After adjusting for key confounding factors such as the case mix index and equipment usage, the association between the intervention and the decrease in HAI rate remained significant, which to some extent enhanced the robustness of the findings, although a causal relationship could not be fully established. These results provide descriptive evidence suggesting a temporal association between adherence improvements and reductions in infection rates; however, causal interpretation should be made cautiously given potential residual confounding and multiplicity considerations. Notably, after Bonferroni correction for multiple comparisons, only the reduction in respiratory infections remained statistically significant, further underscoring the need for cautious interpretation of subtype-specific findings.

The findings highlight the practical utility of the CFIR framework in infection prevention. Although CFIR has been applied in nursing and public health studies (14, 27), few investigations have explicitly integrated it into hand hygiene interventions. By systematically addressing barriers across domains such as intervention characteristics, organizational context, individual attributes, and implementation processes, the present study was associated with improved compliance and concurrent reductions in infection rates. Compared with interventions limited to education (30), feedback (31, 33), or electronic monitoring (32), the multidimensional CFIR-based approach appears to generate broader and more sustainable effects.

Although overall compliance approached recommended standards (21), performance at high-risk moments remained inadequate—for example, compliance before patient contact was only 50.03%, markedly lower than at other times. This highlights a process-oriented, mechanistic approach in practice, which creates vulnerabilities at key checkpoints. Notably, significant differences were observed across job categories: nurses showed better compliance than physicians, while support staff scored lowest. This reflects the insufficient coverage of non-professional staff within existing monitoring systems. Although this study shows that healthcare workers generally hold positive attitudes, the persistent gap in compliance suggests that future research should explore other barriers to the attitude-to-behavior translation, such as environmental cues or ingrained habits. Previous research has explored potential non-linear relationships between hand hygiene compliance and infection outcomes; however, such relationships were not formally tested in the present study (9–11). Future interventions should therefore prioritize execution quality at key junctures rather than merely increasing overall frequency.

This study was conducted at a single center, limiting the representativeness, and generalizability of the findings. The absence of a concurrent control ward or hospital makes the findings potentially susceptible to secular trends, seasonal variation, and regression to the mean. Compliance data were collected through non-announced direct observation; although efforts were made to minimize observer effects, behavior modification due to awareness of monitoring (Hawthorne effect) cannot be entirely excluded. Hand hygiene opportunities were clustered within individuals and clinical units. Because individual identifiers could not be recorded due to ethical restrictions, clustering could not be fully accounted for in the statistical analyses. As a result, standard errors may have been underestimated, and *p*-values should be interpreted cautiously. Although the proportion of missing questionnaire data was below 1%, mean imputation may have introduced minor bias in variance estimates. Formal assessment of measurement equivalence across professional groups was not conducted, which may limit strict comparability of knowledge and perception scores between staff categories, particularly for support staff whose educational background and language comprehension levels may differ from those of physicians and nurses. In addition, questionnaire responses were not linked to specific observed individuals; therefore, no individual-level association analysis between attitude scores and observed hand hygiene compliance was performed. Although thematic stability was observed in the qualitative phase, the relatively small number of interviews across heterogeneous professional roles—particularly within support staff categories—may have limited the depth of role-specific insights. In addition, the relatively small number of support staff participants in the questionnaire survey (*n* = 31) may have reduced statistical stability for subgroup comparisons, and findings related to this group should therefore be interpreted cautiously. Confounding factors such as antimicrobial use, comorbidities, case-mix, and device utilization were not fully controlled. Although multivariable adjustment was performed for selected patient-level and structural indicators, residual confounding cannot be excluded. In addition, other routine infection prevention and quality improvement activities continued during the study period, and their potential influence on HAI trends cannot be fully ruled out. The intervention phase (April–September 2025) was relatively short, which may have influenced observed infection patterns. In addition, the intervention phase (April–September 2025) was relatively short, which may have influenced observed infection patterns. Therefore, the reductions in HAI rates should be interpreted as associations within this context rather than definitive causal effects. Future research should extend these findings through multi-center studies, longer follow-up periods, and more rigorous adjustment for temporal and organizational confounders. By embedding CFIR-guided approaches into routine practice, hospitals may transform hand hygiene from a formal compliance indicator into a substantive safeguard for patient safety.

## Conclusion

This study demonstrated that hand hygiene compliance varied significantly by role and moment, with support staff and pre-patient contact representing the weakest points. Implementation of a CFIR-guided, stratified intervention was associated with improvements across all groups, with pronounced gains among support staff and at high-risk moments, and was associated with reductions in hospital-acquired infection rates. The findings carry important clinical implications. Infection control strategies should focus not only on overall compliance but also on adherence at critical moments. Role-specific interventions are essential: while physicians and nurses may benefit from enhanced monitoring and feedback, support staff require simplified, practical, and supervised training. Furthermore, institutional mechanisms such as structured feedback, adequate provision of hygiene resources, and cross-departmental collaboration are critical for ensuring sustainability.

## Data Availability

The original contributions presented in the study are included in the article/Supplementary material, further inquiries can be directed to the corresponding authors.
