# Supplementary material for: Hand hygiene compliance among healthcare workers before and after a CFIR-guided role-stratified intervention: a mixed-methods study in a tertiary hospital
Source: Front Public Health. 2026 Apr 20;14:1750206. doi: 10.3389/fpubh.2026.1750206 (PMC13136248; doi:10.3389/fpubh.2026.1750206)
Supplement: Supplementary file 1 [file Table_1.docx]

**Supplementary Table 1. Detailed Intervention Measures Based on the Five CFIR Domains**

| **Domain (CFIR)** | **Measures & Description** |
| --- | --- |
| I. Intervention Characteristics |  |
| ① Standardized procedures | Retraining on WHO’s “Five Moments for Hand Hygiene” and six-step handwashing method; clarifying responsibilities and execution points by position. |
| ② Visualized workflow charts and reminder cards | Development of workflow charts and reminder cards displayed at hand hygiene stations. |
| ③ Establishing compliance assessment indicators by position | Setting reasonable compliance rate targets for various positions, incorporated into quality control assessment. |
| ④ Establishing an onboarding assessment system for the logistics department | Assessing supervisors at all levels of logistics. |
| II. Outer Setting |  |
| ① Drawing on national standards and literature indicators | Benchmarking against *Regulations on Hospital Infection Management*, *Hand Hygiene Technical Specifications*, etc. |
| ② Introducing a joint inspection mechanism (quality control, nursing, and logistics departments jointly inspecting) | Monthly spot checks and feedback to promote motivation for improvement under external pressure. |
| ③ Conducting hand hygiene microbial cultures for personnel in key departments, reporting results, and requiring rectification and re-culturing for non-qualified cases until qualified | Monthly feedback of microbiological culture results to promote external supervision and improvement. |
| III. Inner Setting |  |
| ① Multi-level training | Layered training for doctors, nurses, and service staff, with targeted content design; for service staff, adopting on-site practice, videos, and other teaching methods. |
| ② Establishing a feedback and notification mechanism | Monthly disclosure of compliance rates of each department, with incentives for excellent performance. |
| ③ Ensuring sufficient supply of hand hygiene materials | Inspecting and replenishing hand sanitizers, sensor-based handwashing devices, quick-drying hand disinfectants, and other facilities. |
| IV. Characteristics of Individuals |  |
| ① Enhancing risk awareness | Carrying out warning education combined with real cases to improve willingness for conscious execution. |
| ② Distributing knowledge questionnaires + instant feedback | Strengthening knowledge mastery and enhancing consistency between cognition and behavior. |
| ③ Joint management by medical staff | Establishing a “Hand Hygiene Supervisor” system, implementing mutual inspection and evaluation. |
| V. Implementation Process |  |
| ① Appointment of intervention leaders | Setting up hospital infection monitors in each department for unified coordination. |
| ② PDCA cycle promotion | Establishing a continuous quality improvement closed loop. |
| ③ Monthly monitoring data feedback | Including compliance rate, correctness rate, infection rate, to quantify intervention effects. |

# **Supplementary Table 2. Example of Monthly Hand Hygiene Audit and Feedback Report Template**

| **Ward** | **Professional Group** | **Observed Opportunities (n)** | **Compliance Rate (%)** | **Correctness Rate (%)** | **Moment 1 Compliance (%)** | **Identified Key Gaps** | **Performance Category** | **Recommended Corrective Actions** |
| --- | --- | --- | --- | --- | --- | --- | --- | --- |
| Internal Medicine A | Physicians | 135 | 72 | 84 | 58 | Low compliance before patient contact | Improvement Needed (60–79%) | Reinforce bedside reminders; peer-led discussion during rounds |
| Surgery B | Nurses | 210 | 85 | 91 | 83 | Occasional missed moments after environmental contact | Acceptable (≥80%) | Maintain current supervision; highlight best practice examples |
| ICU | Nurses | 160 | 82 | 90 | 75 | Suboptimal compliance during emergency procedures | Improvement Needed (<85% ICU target) | Bedside alcohol rub optimization; rapid-cycle coaching |
| Surgery C | Support Staff | 98 | 55 | 48 | 42 | Poor recognition of hand hygiene opportunities | Targeted Action (<60%) | On-site demonstration; simplified workflow; retraining session |
| Internal Medicine D | Support Staff | 120 | 62 | 53 | 50 | Incomplete technique execution | Improvement Needed (60–79%) | Hands-on retraining with supervision; visual cue reinforcement |

Compliance Rate = (Number of hand hygiene actions performed / Observed opportunities) × 100%.

Correctness Rate = Percentage of performed hand hygiene actions meeting WHO technique standards.

Performance thresholds: ≥80% = Acceptable 60–79% = Improvement Needed <60% = Targeted Corrective Action Required

ICU wards apply a higher target threshold of ≥85% due to elevated infection risk.

Moment 1 refers to “Before patient contact” according to the WHO Five Moments framework.

Data shown are illustrative and serve as a structural example of the feedback report format.

### **Supplementary Table 3. Comparison of Hand Hygiene Opportunities (Structure) Before and After Intervention**

| **Characteristic** | **Pre (n=3,907)** | **Post (n=5,860)** | **χ²** | **P-value** |
| --- | --- | --- | --- | --- |
| Professional Role |  |  | 1.84 | 0.398 |
| - Physicians | 1,180 (30.2%) | 1,712 (29.2%) |  |  |
| - Nurses | 2,340 (59.9%) | 3,595 (61.3%) |  |  |
| - Support Staff | 387 (9.9%) | 553 (9.4%) |  |  |
| Ward Category |  |  | 1.32 | 0.517 |
| - Internal Medicine | 1,605 (41.1%) | 2,360 (40.3%) |  |  |
| - Surgical Wards | 1,510 (38.6%) | 2,325 (39.7%) |  |  |
| - ICUs | 792 (20.3%) | 1,175 (20.0%) |  |  |

## P-values were calculated using the Chi-square test to evaluate the structural comparability between the two phases. P > 0.05 indicates that the baseline characteristics of the observed opportunities were balanced.

## **Supplementary Table 4. Multivariable Poisson Regression Analysis of Factors Associated with HAI Incidence**

| **Variable** | **Adjusted IRR** | **95% CI** | **P-value** |
| --- | --- | --- | --- |
| Post-intervention period (vs. Pre) | 0.62 | 0.50–0.77 | <0.001 |
| Case Mix Index (CMI) | 1.05 | 0.88–1.25 | 0.562 |
| Urinary catheter utilization rate | 1.01 | 0.97–1.05 | 0.421 |
| Central venous catheter utilization rate | 1.02 | 0.98–1.06 | 0.315 |
| Ventilator utilization rate | 1.03 | 0.99–1.08 | 0.148 |
| Surgical volume (per 100 cases) | 1.00 | 0.99–1.01 | 0.844 |
| ICU patient-day proportion | 1.04 | 0.96–1.12 | 0.287 |

Abbreviations: HAI, hospital-acquired infection; IRR, incidence rate ratio; CI, confidence interval; CMI, Case Mix Index.

The model was adjusted for monthly case mix index, device utilization rates, surgical volume, and proportion of ICU patient-days. The natural logarithm of total patient-days was included as an offset variable.
