# Supplementary figures and images for: Hand hygiene compliance among healthcare workers before and after a CFIR-guided role-stratified intervention: a mixed-methods study in a tertiary hospital
Source: Front Public Health. 2026 Apr 20;14:1750206. doi: 10.3389/fpubh.2026.1750206 (PMC13136248; doi:10.3389/fpubh.2026.1750206)

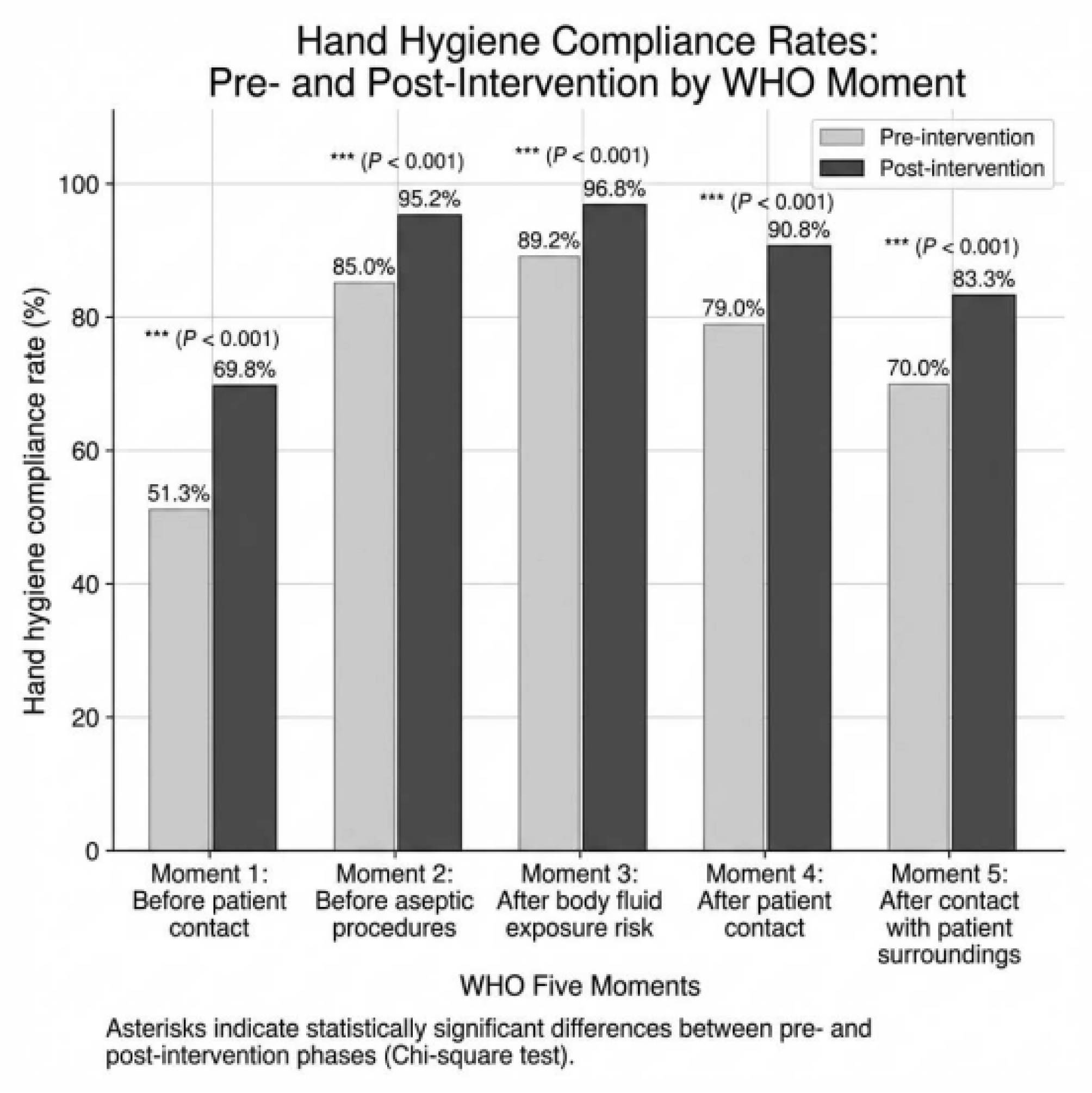

Supplement: Supplementary file 2 [file Image_1.tif]
